# Editorial: Mortality Risk and Related Adverse Outcomes Following Discharge From Inpatient Psychiatric Care

**DOI:** 10.3389/fpsyt.2021.763342

**Published:** 2021-09-27

**Authors:** Merete Nordentoft, Eric D. Caine, Roger T. Webb

**Affiliations:** ^1^Mental Health Centre Copenhagen, Mental Health Services in the Capital Region, Department of Clinical Medicine, University of Copenhagen, Copenhagen, Denmark; ^2^University of Rochester Medical Center, Rochester, NY, United States; ^3^National Institute for Health Research Greater Manchester Patient Safety Translational Research Centre, Centre for Mental Health & Safety, The University of Manchester, Manchester, United Kingdom

**Keywords:** suicide, discharge, mental illness, prevention, mortality, psychiatry

As emphasized by recent papers in *Frontiers in Psychiatry*, (cites) the suicides of persons recently discharged from psychiatric hospitalization remain among the most distressing and challenging events that confront our field. We consider in this commentary many of these challenges and call for a new generation of studies.

## Post-Discharge Suicides Are Harrowing and Complex

Except from the few patients who discharge themselves against medical advice from inpatient stays in mental health facilities, a clinician will have evaluated the discharge plan as safe.

Therefore, post-discharge suicides are usually associated with grief and anger among relatives, frustration, self-blame, sorrow and insecurity among clinicians, and initiatives to improve safety procedures in services among leaders of mental health services. Following the discussions that arise on social media after a post-discharge suicide reveals the depth and intensity of the frustration and despair that is felt by other patients and by relatives. Often, they complain that admission wasn't helpful, that the condition was more or less unchanged between admission and discharge, or that the patient was discharged without sufficient help afterwards.

## Living Back in the Community Is Especially Challenging and Risky

Being admitted to inpatient psychiatric care usually constitutes a medical emergency, reflecting both a person's deteriorating state of mind and the inability of local resources to provide adequate care in the community. Whilst hospitalization may help to resolve acute symptoms, it frequently is insufficient to repair deep social strains; returning to life in the community may be a particularly challenging and risky transition that poses a major threat to patient safety. Examples of such challenges could be unpaid bills, inability to maintain a home in a proper condition, threats of eviction, job loss, conflicts with loved ones or neighbors (including legal restraining orders that forbid contact), inability to recreate meaningful daily life routines and activities, recurring use of alcohol and drugs, difficulties obtaining needed medication, and lack of follow-up from health and mental health services.

## Post-Discharge Suicide Is an Important Public Health Problem

Suicide risk is especially raised during the beginning of inpatient stay and shortly after discharge (1–4). Suicide is an important public health problem, responsible for 800,000 deaths worldwide every year. Furthermore, suicides account for a large proportion of all potential years of life lost in a population (5, 6). In the field of suicide prevention, post-discharge suicide is among the most important problems because it affects a large risk group, and compared to the general population the post-discharge suicide rate is 2–400 times higher, with rates for men above 4,000 per 100,000 during the first post-discharge week (3). Such rates are higher than in any other well-known risk group. Risk of dying, and especially so by suicide, is most acutely raised during the period immediately after and within 3 months of discharge (4). Previously published studies have reported absolute and relative risk estimates for all- cause mortality and for specific causes of death, stratified according to diagnostic category, substance misuse comorbidity and sociodemographic factors, with suicide being the most frequently investigated cause-specific mortality outcome.

In terms of population attributable risk, suicides that occur during the first week post discharge have been estimated as constituting 5 percent of all cases that occur in Denmark, with the population attributable fraction being 15 percent for suicides happening within 3 months of discharge (3). Similar fractions could be expected in other countries with universal free access to admission in mental health services.

Despite concern about post-discharge fatalities, relatively few studies have evaluated temporal trends in post-discharge suicide rates. Analyses of national registry data from Denmark indicate that the rates fell slightly between 1995 and 2016, although remaining unacceptably high since (3). These rates should be a mandatory component of assessing the quality of mental health services. However, many countries currently lack accessible data at a national level for monitoring the risk year by year. It would be ideal to have robust research evidence from a broader spread of countries to develop more comprehensive international evidence. Greater evidence is also needed in relation to a broader range of adverse outcomes other than cause-specific mortality, including non-fatal self-harm, serious accidental injury, and perpetration of and victimization by interpersonal violence (7).

Therefore, it is of utmost importance to develop methods to reduce the elevated risk of suicide post discharge. They are responsible for a substantial proportion of all suicides and constitute an important public health problem. We have focused on this topic in a special issue of *Frontiers in Psychiatry* with a collection of seven papers. In a review of risk of suicide in mood disorders in the Nordic countries, Isometsä confirmed that the relative risk of suicide was consistently found to be especially raised during the first post-discharge weeks, thereafter declining over time but still fivefold higher than in the general population after 5 years. Raised risk also was confirmed in a smaller Norwegian study of patients discharged from acute psychiatric ward (Prestmo et al.).

Results from the UK's National Confidential Inquiry into Suicide and Safety in Mental Health, reported in this issue by Bojanic et al. indicate that discharged patients diagnosed with personality disorder and former inpatients who self-discharged were more likely to die by suicide within a few days after leaving hospital, especially by jumping from high buildings or in front of moving vehicles. Based on their findings, the authors recommend home visits within 2 or 3 days after discharge. In the Danish SAFE project, Madsen et al. found that 5 percent were classified as being at increased risk of suicide when evaluated at a home visit carried out within 1 week after discharge.

In a large study, based on almost 400,000 short-term admissions at the US Veterans Health Administration, Kessler et al. applied machine learning on medical record data, thereby developing a tool with high sensitivity and specificity. With this tool, the authors identified the 5 percent of the discharged population who should be the target group for an intensive case management program—it accounted for between 22.4 and 32.2 percent of the suicides during first year after discharge. Similarly, Senior et al. used a machine learning technique that recognized concepts in free-text of medical records to create the OXmis risk prediction tool.

Tyler et al. described a co-design process involving both staff members and patients in which 4 important initiatives were identified as the most feasible: a multi-agency Discharge Team with a discharge coordinator, inclusive technology-enabled team meetings, universal documentation, and a patient generated discharge plan. All of these elements require testing.

## Risk Evaluation–Is It Useful?

It is difficult for clinicians to decide when it is safe to discharge a patient. More granular research findings are needed in relation to the antecedents of elevated risk post discharge. Most clinicians tend to focus on the individual's apparent mental state prior to leaving.

However, external contextual factors may be just as important. Future research needs to assess the pathways by which discharged patients seek and receive health services and support from other public agencies. Discharge planning strategies must develop new methods for rigorously evaluating family, social, and vocational factors that may have direct impacts on recently discharged patient—the clinical presentation seen in hospital may be subject to rapid change when facing the negative personal consequences of past actions and lost connections.

There has been much discussion and debate about the usefulness of risk prediction. Scales have been criticized for their low positive predictive values, which is an uninformative measure, given that suicide is so rare in absolute terms—even in this exceptionally “high-risk” group. This argument may be misplaced, however. Are there persons, having just been discharged from a psychiatric inpatient facility, for whom careful follow-up is not warranted? The real danger lies in overlooking apparent false negatives, whereby clinicians may assume that persons who seem “fine” symptomatically when leaving hospital will remain stable in the face of prior exacerbating psychosocial factors that were never resolved during their inpatient stay. It has been argued that the clinical evaluation should be done instead of overreliance on risk scales. However, clinical evaluation also can be uncertain when done haphazardly or when it does not include an appraisal of the patient's fuller life situation. As a prophylactic intervention, should hospitals undertake preventive home visits shortly after discharge for persons in selected high-risk groups or for all discharged patients?

We understand that suicide prevention among persons recently discharged from inpatient care poses vexing challenges. Home visiting, for example, may be costly and viewed as inefficient if all eligible persons are seen. Yet, if we adopt an approach that seeks only to contact persons viewed as being “high-risk,” it is likely that the population impact will be small (8). However, we have been learning during the COVID-19 pandemic that there are many ways to establish contact with patients, including telehealth checks and online web connections that use mobile apps.

It is timely to consider new approaches to staying connected with individuals who have been discharged recently. To test effectiveness and possible adverse effects of implementing different interventions, very large randomized controlled trials of the most promising solutions should be carried out. With the availability of new technologies, we may be able to show convincingly that many post-discharge suicides are preventable.

## Author Contributions

MN drafter the first version of the manuscript. RW and EC revised the manuscript. All authors approved the final version.

## Conflict of Interest

The authors declare that the research was conducted in the absence of any commercial or financial relationships that could be construed as a potential conflict of interest.

## Publisher's Note

All claims expressed in this article are solely those of the authors and do not necessarily represent those of their affiliated organizations, or those of the publisher, the editors and the reviewers. Any product that may be evaluated in this article, or claim that may be made by its manufacturer, is not guaranteed or endorsed by the publisher.
